# Supplementary material for: Prenatal environmental determinants of aromatase brain-promoter methylation in cord blood: chemical, airborne, pharmacological, and nutritional factors
Source: Environ Epigenet. 2026 Aug 12;12(1):dvag027. doi: 10.1093/eep/dvag027 (PMC13525341; doi:10.1093/eep/dvag027)
Supplement: Supplementary file 1 [file dvag027_supplemental_files.zip › response_to_reviewer_comments_R1.docx]

**Response to Reviewers**

**Manuscript ID:** ENVEPIG-2025-036

**Title:** Prenatal Environmental Determinants of Aromatase Brain-Promoter Methylation in Cord Blood: Chemical, Airborne, Pharmacological, and Nutritional Factors

**Journal:** *Environmental Epigenetics*

**Reviewer 1**

**General Comments**

**Comment:** *This manuscript explores the relation between 25 markers reflecting exposure to plastics, air pollutants, pharmacological factors, nutrition, and sunlight and methylation at the brain-specific CYP19A1 promoter region. Individual and mixture effects of the exposures are examined, and the authors observe a positive association. Overall, the manuscript is well-written and concise and includes a balanced introduction rooted in biology, a clearly communicated result section, alongside a discussion that highlights the biological significance of the findings. My specific comments are below but broadly are related to the mixture methods and examining methylation over a window. Throughout the review, I have tried to follow the page numbers from the original document (aka those on the inner side, near the text) and not the ones added on the outer edge of the margin when the PDF was created. Hope they make sense. Thank you so much for your dedicated hard work and important contribution to science!*

**Response:** We thank the reviewer for the insightful and constructive comments on the manuscript. Their positive and collaborative tone throughout made this review a pleasure to read.

**Abstract**

**Comment:** *1. Suggest reporting effect estimates in 2 decimal places unless the authors have a strong reason that suggests the data is so powerful for us to be sure till the 3rd.*

**Response:** Thank you. We have changed the reporting of effect sizes to two decimal places throughout the manuscript.

**Comment:** *2. Line 35/49: Sorry if I misunderstood, but please clarify the biomatrix in which methylation of the CYP19A1 P1.f promoter was measured.*

**Response:** DNA methylation (DNAm) of the *CYP19A1* P1.f promoter was measured in umbilical cord blood (Methods page 11, lines 334-44) using the Illumina MethylationEPIC (850k) array. We evaluated DNA methylation over P1.f using an “expanding window” approach, beginning with a 7-CpG window directly on P1.f and then expanding this window to include the upstream promoter region to determine the specificity of effect for P1.f. This is the approach taken in the larger *Nature Communications* study for which this manuscript is a follow-up.

**Introduction**

**Comment:** *1. Lines 45-46, 48-50: Kindly suggest rewording the first paragraph. While I appreciate the team’s nuanced discussion, personally, the framing of this paragraph feels to strong. I am unsure of how confident and certain we are about how much awareness, genetics, and environmental changes each contribute to this increase. I’d suggest saying something like “While genetics and increased awareness, changes in diagnostic criteria, and its accessibility partly are why we observe the increase in incidence, the role of the environment is equally important to consider as per the Dohad hypothesis.” That being said, I acknowledge this suggestion is very much dependent on personal writing preferences and culture, but I would recommend the authors be considerate about the science communication and public message in the first paragraph.*

**Response:** We thank the reviewer for this thoughtful suggestion regarding the framing of our opening paragraph. We have now modified the introduction to better reflect this complexity: “...The rise in autism is only partly accounted for by changes in diagnostic criteria and improved awareness, and the short time window suggests it cannot be explained by genetic changes. Modern environmental exposures are therefore important to consider.” (page 2, lines 47-49) We include a citation for the *Developmental Origins of Health and Disease* (DOHaD), as per the reviewer’s helpful suggestion.

**Comment:** *2. Line 53: “First, autism is diagnosed considerably more often in males than in females”. I appreciate the authors mention of this but I’d kindly partly disagree with this. As the authors, point out earlier, this is partly due to the imperfections in diagnostic evaluations, the higher rate of misdiagnosis among females, historical disparities in accessibility of diagnosis and our understanding of how autism differently presents among non-male gender based on gender roles, among others factors that lead to why there is more “diagnosis”. Now, yes of course, biological susceptibility could be part of this story, but I am not confident if we can parse out the attributable factors for each of these different factors. I suggest the authors to either exclude this sentence. Their beautiful justification in the following paragraph comes at this point again but with more biological significance, which is nice!*

**Response:** We appreciate the reviewer’s nuanced perspective and excellent points regarding the complexities of autism prevalence. While we note that the central role of endocrine-related mechanisms in the aetiology of autism suggests that the higher proportion of male diagnoses may, at least in part, reflect genuine biological susceptibility, we have now acknowledged the reviewer’s point with the following caveat: “First, while there is debate around why,^1,2^ autism is diagnosed considerably more often in males than in females.” (page 2, line 53)

To further address the broader issue of sex differences, we have now repeated the WQS regression analysis stratified by sex to examine whether the association between the prenatal environmental mixture and P1.f methylation differs in males vs. females. These new findings are detailed in the revised **Results** (page 7, lines 198-200) and further discussed in our response to **Reviewer 2, Comment 4**.

**Comment:** *3. I’m not an aromatase expert, but is it only coded by one gene, and are there no effects of others through any feedback or networks?*

**Response:** Yes, the aromatase protein is encoded by a single gene, *CYP19A1*. We now state this explicitly in the **Methods** (page 11, lines 338-39). As the reviewer suggests, aromatase also operates within complex developmental signalling networks. We have further clarified these relationships in the revised manuscript:

- **Upstream Regulation:** *CYP19A1* expression is controlled by multiple transcription factors that bind at tissue-specific promoters. For example, estrogen receptor alpha (ERα) binds specifically at the brain-specific P1.f promoter, which is the focus of this study.
- **Downstream Networks:** Once expressed, aromatase influences many endocrine-related signalling pathways, including those involving key neurodevelopmental factors such as *RORA* and *BDNF*.

Importantly, our previous study using both human cohorts and mouse models indicates that within these broad networks, *CYP19A1* represents a primary point of susceptibility where environmental signals (such as bisphenols) converge to influence autism likelihood.

We have now expanded the **Discussion** (page 8, lines 259-63) to provide further information on aromatase-related gene networks, specifically addressing the reviewer’s query regarding feedback loops.

**Comment:** *4. Line 71: please add citation for BPA x autism phrase.*

**Response:** Thank you. We have now added supporting citations (page 2, line 72).

**Comment:** *5. Line 74-76: is this concordance higher than any other tissues, if they have been evaluated?*

**Response:** We did not evaluate additional tissue types in the present study. Our rationale (lines 74–76) is based on established evidence that *CYP19A1* exhibits high blood-brain concordance. While tissues such as buccal swabs are also common proxies in human cohorts, the reliability of any surrogate tissue is somewhat dependent on the specific genomic region in question. Given that *CYP19A1* demonstrates strong cross-tissue correlation at the promoter of interest, we argue that the cord-blood data employed here provides an appropriate proxy for brain DNAm.

**Methods**

**Comment:** *1. I apologize if I missed this but were the twins retained in the study? Can the authors share some insight on whether the inclusion of twins in a methylation study would require different methods than when analyzing methylation in a cohort with singletons?*

**Response:** We clarify that twins were excluded from the final analysis. While we acknowledge that the inclusion of a small number of twin pairs (here, <1% of the cohort) would be unlikely to influence the overall mixture weights or effect sizes, we elected to remove them to ensure strict independence of observations. Had they been retained, a mixed-effects model with a random intercept for Family ID (or Generalized Estimating Equations (GEE)) would have been required to account for within-pair correlation.

**Comment:** *2. Please add LODs and detection rates for all environmental exposures quantified (e.g., bisphenols, phthalates, and air pollutants). These exposures are likely to be skewed as well. Though they are operationalized as categorical variables, I kindly suggest the authors to consider reporting the geometric mean and SDs. Also, please consider tabulating the dichotomized version of these exposures that were used in the models so that readers can understand the distribution of detects vs non-detects, etc.*

**Response:** We thank the reviewer for these suggestions. We now present LODs and detection rates for all quantified environmental exposures, i.e. the phthalates and bisphenols (air pollution was estimated from geographical data) in a new **Supplementary Table 3**. In Table 1, we provide the median and interquartile range (IQR) for continuous exposures. While we acknowledge the reviewer’s suggestion for geometric means, we believe the median/IQR is more appropriate here as it directly informs the dichotomization at the median used in our primary analyses. This ensures consistency between our descriptive data and our modelling approach. Regarding the distribution of "high" vs "low" exposures, we have now clarified in the Table 1 caption that because dichotomization occurred at the median, the "high" category consistently represents 50% of the sample size (N) reported for each variable.

**Comment:** *3. WQS vs other methods for the mixture analysis: A drawback of WQS is that WQS requires the researcher to pre-specify a direction of the effect for all components of the mixture. WQS tests which component contributes the most to the total effect that was pre-specified. Could the authors please share why they chose WQS over other methods? Why is running WQS two ways as shown in Supplement better than running other methods that would allow interactions and any effect direction? I recommend the authors include a secondary analysis with qgcomp which allows each exposure to have positive or negative effects (aka we don’t assume the effect direction) and then calculates the combined effect due to all the components without us pre-specifying it. Alternatively, one can consider BKMR to examine interaction or dose-response effects on methylation, especially if the analysis is targeted.*

**Response:** We thank the reviewer for this thoughtful critique of the statistical approach used here. The rationale for employing WQS regression in this setting is that the method is well established in environmental mixture studies and more immediately interpretable than most other methods, providing a simple mixture “index” variable, with a clear weight assigned to each exposure. While BKMR, as a Bayesian approach, is indeed more flexible and mathematically sophisticated, allowing for non-linear effects and interactions between exposures, it is also computationally intensive and performs poorly when the exposure set exceeds 15-20 variables in a study of our size.

We acknowledge the debate around the advantages and disadvantages of WQS regression and quantile g-computation (QGC),^3-5^ including the fact that QGC does not require assumptions of directionality. However, we would also note that

1. QGC estimates the net effect of increasing all exposures on their original scales and therefore allows protective and harmful components to offset one another. In contrast, our WQS regression analysis was designed to estimate the joint effect of increasing exposure to autism-related risk factors by orienting each exposure in the hypothesised risk direction. Accordingly, it addressed a different research question, that is, whether greater cumulative burden of autism-relevant environmental risk factors was associated with higher methylation of the *CYP19A1* P1.f promoter and if so, what was the relative contribution of individual components.
2. The directionality constraints imposed in this study were informed by prior studies (e.g. high, not low, prenatal BPA exposure is consistently linked to increased autism symptoms), and
3. Incorrectly encoded exposures will bias the WQS regression model towards the null by diminishing the overall mixture effect on the outcome; the significant mixture effect identified here therefore cannot be explained as an artefact of miss-specified exposures, instead reflecting a conservative estimate of the likely true association.

We appreciate the reviewer’s perspective, however, and have modified the **limitations** section accordingly to acknowledge quantile g-computation as well as BKMR as useful alternative methods (page 9, lines 283-95).

**Comment:** *4. Could the authors share more about why they chose to look at a CpG window instead of individual CpGs? I understand an average methylation may give an insight into overall methylation at the promoter region or to a point, a cumulative effect. This idea may also be captured by multivariate regressions. A secondary analysis to understand which specific CpGs are targeted might also be interesting. Also, look forward to an untargeted EWAS related to this! This is great work!*

**Response:** The analysis of a CpG window rather than individual sites was guided by both biological and statistical considerations. DNA methylation typically regulates gene expression through coordinated changes across functional genomic regions (e.g., promoters) rather than at CpG sites in isolation. By evaluating the P1.f promoter as a single regulatory unit, we better capture the functional signal that influences transcription factor binding and gene silencing or activation, improving power to detect subtle, consistent shifts that might not reach significance at the level of individual CpG sites while simultaneously reducing the multiple testing burden. Importantly, this region-based approach was the method used in our prior *Nature Communications* study (Symeonides et al., 2024) for which this study is a follow-up.

We agree with the reviewer that a non-targeted EWAS evaluating the relationship between this prenatal environmental mixture and cord-blood DNA methylation would indeed provide a fascinating future study.

**Results**

**Comment:** *1. Line 173: there is a special character in front of the word "threshold" which is not printing properly. Please check and/or add a description of this criteria.*

**Response:** Thank you for highlighting this—it is the Greek letter tau (**ꞇ**). We will proof the uploaded submission to ensure that the typo is corrected.

**Discussion**

**Comment:** *1. The present analysis is commendable, and I congratulate the authors for taking a dive into it! Quick question: in relation to the promoter region of CYP19A1, are there any feedback loops, networks, or co-regulation to consider? If so, these can be future directions to explore and discuss in the Discussion section.*

**Response:** We thank the reviewer for these positive comments. We have now expanded the discussion as suggested and as explained further in response to the reviewer’s **Introduction—Comment 3**.

**Comment:** *2. Great work! I kindly request the authors to please further discuss the exposure-based limitations of this analysis. For example, a limitation of this study is that it only considers a selection of exposures from our exposome. There could also be residual confounding. Also, some exposures were measured twice, whereas some were not, which could influence mixture effect estimation of each exposure class. The temporal variation of exposures during pregnancy and its variable effects on methylation during pregnancy remain to be explored.*

**Response:** We thank the reviewer for these points and have updated the **limitations** section accordingly (page 9, lines 283-95).

**Comment:** *3. Some exposure classes (e.g., air pollution, plastics, UV) were measured more than once during pregnancy, and some were not. This means that the WQS mixture does not have an equal number of components representing each biomarker of exposure. Further, each exposure class does not have an equal number of biomarkers that represent it (e.g., Box 1 notes that “nutrition and sunlight” have n = 4 markers while “plastics” have n = 11). How are the authors accounting for this unequal representation in WQS? Does the number of sampling timepoints for some marker lead to over-representation of a marker (or class) and so more effect being estimated to be coming from that marker (or class)?*

**Response:** This is an important consideration. We should first clarify, however, that the bisphenols and phthalates were in fact measured once, at 36 weeks’ gestation. Likewise, air pollution was not measured longitudinally; rather, multiple dimensions of indoor and outdoor pollution were captured (e.g. NO_2_ and PM_2.5_, mould and/or dampness). The one exposure measured twice was ultraviolet (UV) radiation, assessed in trimester 1 and 2. This is because, due to seasonal shifts, UV measured in each trimester captures a unique exposure profile (e.g. summer vs. winter). Importantly, WQS regression is designed to handle multicollinearity, meaning that the method is well suited to analysing classes of closely related exposures, such as the metabolites of DEHP (MEOHP, MEHHP, and MECPP). Moreover, to address the reviewer’s concern regarding over-representation of some classes, we note that—while it is true that larger classes have more “opportunity” to be selected—WQS regression assigns weights for a given exposure independently of other exposures in the same class. Nor does WQS regression as implemented here assign class-level effects (i.e. it is not hierarchical).

We thank the reviewer again for these insightful and positive comments, which were a pleasure to read and have provided us the opportunity to enhance the clarity and methodological rigour of the manuscript.

**Reviewer 2**

**Comments to the Author**

**Comment**: This is a very interesting study of how environmental risk factors for autism are related to methylation of the aromatase gene in cord blood.  Strengths of the study include its sample size, variety of exposures examined, and mixture approach.  However, these different variables were measured with varying precision and ultimately it appears that in order to use WQS, many detailed variables had to be dichotomized (i.e. reduced in precision).  I feel this is a major limitation and reduces the interpretability and impact of the findings.  However, this is an important research question and I’ve suggested some ways the authors might consider re-framing parts of the analysis.

**Response**: We thank the reviewer for their positive and constructive comments and critiques.

**Major Comments**

**Comment:** *1. The missingness for some of the included variables is quite high. For example, mold and heating are 45%. This raises concerns about the validity of the MICE imputation. Given that mold had one of the larger weights in the WQS, I do not have confidence in this finding. I recommend that the authors either remove variables with missingness >30% or give stronger justification for why they are keeping them in the model.*

**Response:** This is a valid query and we thank the reviewer for raising it. We acknowledge the trade-offs in the inclusion of variables with higher missingness, such as mould, while providing the following justification:

1. Multiple imputation by chained equations (MICE) has been shown to yield unbiased estimates even with missingness up to 50% in large datasets.^6^ With a sample size of N > 800 and a high number of auxiliary variables (most with well under 30% missingness) to inform the imputation, our approach conforms to established practice.
2. A key factor in our decision was the operationalisation of these variables. By using dichotomised indicators rather than continuous measures, the model is inherently more resilient to minor imputation errors, as it only requires the participant to be correctly classified as "above or below" a threshold.

Given these considerations, and the fact that mould is an important component of the "biological exposome" with prior links to neurodevelopmental outcomes, we elected to include it. Nonetheless, we have updated the **limitations** section to explicitly state that weights assigned to high-missingness variables should be interpreted with appropriate caution (page 8, lines 284-5).

**Comment:** *2. When the authors say that air pollution levels were measured “at birth”, do they mean day or week? And why was this window chosen? Air pollution has been linked to neurodevelopment in many studies but they have typically examined exposures across the pregnancy and specific weeks/trimesters. However, since the authors are interested in cord blood methylation, it potentially makes sense to examine a more proximate window to birth. It needs better justification in the methods and/or can be discussed as a limitation.*

**Response:** Air pollution (NO2 and PM2.5) were estimated from the mother’s postcode at birth (**Methods**, page 10, line 325-26). This timepoint was chosen, as the reviewer suggests, because it provides a snapshot of pollution exposure at a key pre- and perinatal window (when DNA methylation was also profiled). However, we should also note that postcode-derived estimates are typically more stable than single-timepoint chemical measures, making the precise timepoint less critical.

**Comment:** *3. Given the narrow distribution of methylation levels, it would be helpful for the authors to interpret the adjusted mean difference associated with the chemical exposures. For example, is the AMD effect size of BPA exposure greater in magnitude than an IQR change in the methylation of the 7 CpG window? The WQS mixture effect size is even bigger. It’s hard to wrap my head around these effect sizes given the vary narrow distribution of the methylation data.*

**Response:** This is a helpful suggestion—DNA methylation at the *CYP19A1* P1.f promoter is indeed tightly regulated, with a narrow distribution in cord blood. We have now updated the **Results** (page 6, lines 177-179) to contextualise the estimated mixture effect relative to the cohort's natural variability at the P1.f promoter.

In the BIS cohort, the interquartile range (IQR) for DNA methylation across the 7-CpG P1.f window spans 0.01 beta units (0.40 to 0.41). The estimated mixture effect (AMD_WQS_ = 0.71) is substantially larger than the natural variation observed in the population. Specifically, a full-unit increase in the WQS index (moving from the lowest to the highest exposure profile) is associated with a shift in methylation many times greater than the cohort's IQR.

This indicates that the cumulative environmental burden is linked to a major redistribution of methylation at the aromatase brain promoter. While such large shifts for an individual are unlikely to occur across the entire 25-component exposome, these findings indicate that even a moderate increase in the mixture index is associated with a substantial biological deviation from the cohort norm.

**Comment:** *4. Given that previous study found an association between aromatase and autism only in males, did the authors consider examining sex modification in this analysis?*

**Response:** This is an excellent suggestion. While we did not hypothesise that BPA or the broader prenatal mixture elicit differential effects on DNA methylation of the aromatase P1.f promoter in males vs. females—only that subsequent effects on autism outcomes are stronger in males, as our previous study found—we have now included additional sex-stratified WQS regression analyses to confirm this. These stratified analyses found that the mixture effect on P1.f methylation did not markedly differ in males and females, nor, however, did it reach significance in either subgroup with the reduced sample size. We now include these further findings in the updated **Results** (page 7, lines 195-200).

**Comment:** *5. The 25 factors included this study seem a bit arbitrary. There are many factors that have been tied to autism that are not included (e.g., pesticides and metals) while other factors like mold and UV exposure have limited evidence linking to autism. It would be helpful for the authors to give more justification for the inclusion of these factors and to acknowledge that it is not an exhaustive mixture of risk factors.*

**Response:** We thank the reviewer for the constructive feedback regarding the selection of environmental factors. We agree that no single study can capture the full complexity of the prenatal exposome, and we appreciate the opportunity to clarify the selection criteria.

The 25 factors selected for this study were not intended to represent an exhaustive list of all known autism risk factors, but were chosen based on two primary criteria (**Methods**, page 10, line 317-20):

1. **Prior Evidence of Neurodevelopmental Impact:** Each factor belongs to one of five classes (manufactured chemicals, air pollution, pharmacological, nutrition, and sunlight-related factors) previously linked to neurodevelopmental outcomes or aromatase regulation in epidemiological or mechanistic studies. For example, while the evidence for household mould and UV exposure is emerging, recent studies have suggested they may influence systemic inflammation or vitamin D pathways, both of which are relevant to the "estrogen-androgen" balance hypothesis central to this paper.
2. **Data Availability within the Barwon Infant Study (BIS):** As this study utilizes the BIS birth cohort, we were limited to exposures with high-quality, prospectively collected data. Factors like pesticides and heavy metals—while undeniably important in the broader context of autism research—did not meet the internal data density or quality requirements for the Weighted Quantile Sum (WQS) regression modelling in this specific cohort at this time.

We have now updated the **Methods** (page 10, line 317-20) to explicitly state that the mixture is a representative subset of the prenatal exposome rather than an exhaustive catalogue. We have expanded the **limitations** section (page 9, lines 283-95) to acknowledge the non-exhaustive nature of the mixture, specifically citing heavy metals, and highlight that future studies integrating a broader range of toxicant classes and are necessary to fully map the environmental landscape of aromatase methylation.

**Comment:** *6. A major limitation is the imprecise measurement of many of the exposures. Even the ones that were more precisely measured, like the phthalates and phenols, were dichotomized for the analysis, thus losing important and potentially informative variability in the exposure. Could the authors do a model that includes only the continuous exposures that have finer grain measurements? That way they can actually examine the dose-response association.*

**Response:** We appreciate the reviewer’s perspective on the loss of granularity inherent in dichotomization. However, we chose this approach to maintain the primary objective of the study: expanding beyond just BPA to evaluate the aggregate effect of a diverse, multi-domain exposome on *CYP19A1* brain-promoter methylation. Specifically,

1. WQS regression requires all components in the mixture to share a common scale to accurately calculate weights. Because the mixture incorporates both high-precision biomarkers and critical binary lifestyle factors (e.g., specific medication use), dichotomising the continuous biomarkers was necessary to ensure all 25 factors could be modelled together without scale-induced bias.
2. While a continuous-only model (e.g., using only phthalates/phenols) would allow for dose-response analysis, the inclusion of these binary "lifestyle" factors, is critical for a holistic understanding of the prenatal environment’s impact on *CYP19A1* methylation, which was the core objective of this study.

We include a clarifying statement in the **Methods** section regarding the scaling requirements of WQSr (**Methods** page 11, lines 355-358): “*WQS regression requires all exposures to be on the same scale. Given that some exposures were categorical (yes/no), we transformed all continuous exposures into binary indicators using the classification of above median (or above LOD) vs. the rest*”. Additionally, we have updated the **Limitations** section to acknowledge some of the constraints inherent in WQS regression (page 9, lines 283-95).

**Comment:** *7. Was there any kind of socioeconomic measure included in the model? Is there potential for unmeasured confounding?*

**Response:** We adjusted, in the WQSr model, for cord-blood cell-type proportions, maternal contamination of cord blood, time of day of urine collection, sex, gestational age, birth year, and the mQTL genetic variant rs7181429, which is associated with methylation differences at the P1.f promoter. While the possibility of residual unmeasured confounding cannot be definitively excluded in an observational setting—and we now include this point in the **limitations** section (page 9, lines 283-95)—we have now performed a **sensitivity analysis** additionally adjusting for **household income** to address the reviewer’s specific concern. The association between the environmental mixture and P1.f methylation was of similar magnitude and remained significant (AMD_WQS_ = 0.75, *p* = 0.016). We have updated the **Methods** (page 12, lines 373-375) and **Results** (page 7, lines 195-200) sections to include this further adjustment. We thank the reviewer for this suggestion.

**Comment:** *8. Also important to note as a limitations that the EDCs were measured in a single spot urine, and are thus subject to misclassification.*

**Response:** We acknowledge this limitation while noting that the use of a single-spot sample in this context likely leads to **non-differential misclassification**, which typically biases results toward the null. The fact that we identified a significant association between the environmental mixture and *CYP19A1* methylation despite this potential "noise" suggests that the underlying biological signal is robust. Furthermore, the samples were collected during a critical window of prenatal development (mid-pregnancy), which is theoretically the most relevant period for establishing the epigenetic programming we are investigating.

We have now expanded the **Limitations** section to explicitly state that our exposure assessment is intended to represent a "snapshot" of pregnancy rather than the cumulative chronic exposure throughout the entire gestational period (page 9, lines 285-290).

**Comment:** *9. This may be beyond the scope of this study, but did the authors consider a mediation analysis to evaluate whether aromatase methylation is mediating associations between these factors and autism risk in their sample?*

**Response:** This is an excellent suggestion for future work, but we agree that a formal mediation analysis is beyond the scope of the present study. We have expanded the future directions to raise this point (page 9, lines 293-295).

**Minor Comments**

**Comment:** *10. Please specify whether the postmortem brain study in the introduction included males only or males and females (line 64)*

**Response:** We have updated the text (page 2, line 64) to specify that the autism participants in this postmortem brain study consisted of males only.

**Comment:** *11. In the mice studies mentioned in line 78-80, please specify the sex of the animals included.*

**Response:** We have clarified in the text (page 2, lines 79-80) that these experiments were conducted in male mice.

**Comment:** *12. In other studies comparing methylation between autistic and non-autistic individuals, does the aromatase promoter come up? It seems important to acknowledge previous studies of epigenetic patterns associated with autism.*

**Response:** The CYP19A1 promoter has indeed appeared in previous epigenome-wide association studies (EWAS) for autism. For example, a meta-analysis of two EWAS for autism identified two CpGs in the region specifically around the *CYP19A1* *brain* promoter that were significantly associated (*P* < 0.05) with autism in both studies (https://link.springer.com/article/10.1186/s13229-018-0224-6). We now reference this finding in the revised introduction (page 2, line 76).

**Comment:** *13. Have all the factors examined in this study been linked to autism or related symptoms/traits in the Barwon cohort? Would be helpful to clarify whether the authors are including known or suspected risk factors.*

**Response:** Not all 25 factors have been individually linked to clinical autism in the BIS cohort to date. We have clarified in response to the reviewer’s **comment 5** that these were included as suspected risk factors based on broader neurodevelopmental relevance from prior studies and their potential influence on aromatase signalling, rather than confirmed associations in BIS. Importantly, WQS regression assigns differential weights based on actual relationships identified in the data, meaning that the model is not unduly biased by exposures with less relevance.

**Comment:** *14. What was the detectability of the bisphenols and phthalate metabolites examined in this study? Could this information be included in a supplementary table?*

**Response:** Thank you for this suggestion. The phthalate metabolites used in this study were detectable in 93-100% of participants depending on the specific compound, and the bisphenols in 16-55%. We now include this information in Supplementary Table 3.

**Comment:** *15. The authors dichotomized UV radiation, folate, and air pollution levels at their median value. Rather than call the = median. However, I discourage the authors from dichotomizing at all as I think it would be better to do the analysis with the full range of the continuous data.*

**Response:** We acknowledge the reviewer’s point regarding continuous data while noting again that WQS regression requires a uniform scale for all mixture components. To include the questionnaire-based binary variables (the "exposome" breadth), dichotomisation of continuous variables at the median was required to prevent scaling artifacts in weight distribution.

**Comment:** *16. Could the authors provide some context for the CYP19A1 mQTL. Specifically, what is the “effect" of this allele in prior studies?*

**Response:** In the Whole-Blood mQTL Atlas (<https://susztaklab.com/mqtl/mQTL.php>), the effect allele, A, of this mQTL, rs7181429, is associated with reduced methylation of cg09819222 (estimate = -0.325, p = 6.4x10^-10^) and cg19946085 (estimate = -0.305, p = 2.0x10^-11^), both located at *CYP19A1*’s brain promoter P1.f. We have now added this further information to the **Methods** (page 12, lines 371-375), as requested.

**Comment:** *17. Please cite previous study mentioned on line 146.*

**Response:** Thank you. We have added the citation to our previous study (Symeonides et al., *Nature Communications* 2024).

**Comment:** *18. Line 207: "...multiple environmental exposures act together...". Given the exploratory nature of these findings, I would caution against causal-sounding language. Perhaps say "may act.." "Targeted effect" (line 228) is also overly causal language.*

**Response:** We appreciate this caution and have altered "act together" to "**may** act together" and "highlighting a targeted effect" to "**suggesting** a targeted effect" (page 8, lines 220 and 238).

**Comment:** *19. It is curious that a subset of WQS models had higher weights for SSRIs. However, I feel that the discussion over interprets this as potentially meaningful, given that the overall summary of the weights was below the threshold of interest.*

**Response:** While the subset of models showing higher weights is intriguing and consistent with prior studies on SSRIs, we accept the reviewer’s reasoning and have elected to remove this sentence from the discussion.

**Comment:** *20. How was mold/dampness measured? Was it a yes/no question?*

**Response:** Yes, mould/dampness was measured via a maternal self-report questionnaire and coded as 1 (Yes) or 0 (no). This has been further clarified in the **Methods** (page 10, line 327).

**Comment:** *21. The combination of MICE and WQS with repeated holdout was a clever approach. Does that mean that each dot in Figure 2 is the average of 100 iterations?*

**Response:** Thank you! Yes, that is correct—we have clarified this in the Figure 2 caption (page 7, line 214).

**References**

1.	Baron-Cohen S, Lombardo MV, Auyeung B, Ashwin E, Chakrabarti B, Knickmeyer R. Why are autism spectrum conditions more prevalent in males? *PLoS Biol* 2011; **9**(6): e1001081.

2.	Cruz S, Zubizarreta SC, Costa AD, et al. Is There a Bias Towards Males in the Diagnosis of Autism? A Systematic Review and Meta-Analysis. *Neuropsychol Rev* 2025; **35**(1): 153-76.

3.	Keil AP, Buckley JP, O'Brien KM, Ferguson KK, Zhao S, White AJ. A Quantile-Based g-Computation Approach to Addressing the Effects of Exposure Mixtures. *Environ Health Perspect* 2020; **128**(4): 47004.

4.	Gennings C. Comment on "A Quantile-Based g-Computation Approach to Addressing the Effects of Exposure Mixtures". *Environ Health Perspect* 2021; **129**(3): 38001.

5.	Keil AP, Buckley JP, O'Brien KM, Ferguson KK, Zhao S, White AJ. Response to "Comment on 'A Quantile-Based g-Computation Approach to Addressing the Effects of Exposure Mixtures'". *Environ Health Perspect* 2021; **129**(3): 38002.

6.	Junaid KP, Kiran T, Gupta M, Kishore K, Siwatch S. How much missing data is too much to impute for longitudinal health indicators? A preliminary guideline for the choice of the extent of missing proportion to impute with multiple imputation by chained equations. *Popul Health Metr* 2025; **23**(1): 2.
